# Targeting immune cellular populations and transcription factors: unraveling the therapeutic potential of JQF for NAFLD

**DOI:** 10.3389/fimmu.2024.1445924

**Published:** 2025-01-07

**Authors:** Lijuan Zhou, Jingyi Zhao, Kaile Ma, Rui Hao, Chensi Yao, Xiaowen Gou, Chuanxi Tian, Li Wan, Min Li, Xiaolin Tong

**Affiliations:** ^1^ Institute of Metabolic Diseases, Guang’anmen Hospital, China Academy of Chinese Medical Sciences, Beijing, China; ^2^ Clinical Medical College, Beijing University of Chinese Medicine, Beijing, China; ^3^ Molecular Biology Laboratory, Guang’anmen Hospital, China Academy of Chinese Medical Sciences, Beijing, China

**Keywords:** single-cell RNA sequencing, spatial transcriptomics, non-alcoholic fatty liver disease, macrophages, T cells, traditional Chinese medicine

## Abstract

**Background:**

Non-alcoholic fatty liver disease (NAFLD) constitutes the most prevalent chronic liver disease worldwide. Progression to non-alcoholic steatohepatitis (NASH), the immune cell reservoir within the liver undergoes remodeling, exacerbating liver inflammation and potentially leading to liver fibrosis. Jiangtang Qingre Formula (JQF) is an effective prescription for the clinical treatment of NAFLD. However, its underlying mechanism of action remains unclear.

**Methods:**

Using a high-fat diet-induced NAFLD mouse model, we evaluated JQF’s effects with biochemical tests and histopathology. Single-cell RNA sequencing and spatial transcriptomics furthered our understanding of NAFLD pathophysiology and JQF’s treatment mechanisms.

**Results:**

Our findings initially revealed significant improvements in JQF on hepatic steatosis, inflammation, fibrosis and glucose tolerance in NAFLD mice. Furthermore, significant changes were observed in the immune cells including monocytes, macrophages, and T cells in the livers of NAFLD mice. Notably, regions infiltrated by T cells presented the most severe liver inflammation and fibrosis. Importantly, JQF effectively modulated these immune cells. Advanced subcluster and cell communication analyses identified key macrophage (KCs, MoMFs) and T cell (Tc, Th2) subpopulations in JQF’s therapeutic actions. Further SCENIC analysis additionally uncovered the essential transcription factors that regulate these cell subclusters, such as Stat2, Mta3, Eomes, and Etv5.

**Conclusion:**

Overall, our research suggests a promising potential therapeutic agent and identifies critical cell populations and transcription factors that contribute to its therapeutic effects, thereby revealing potential therapeutic targets for NAFLD.

## 1 Introduction

Non-alcoholic fatty liver disease (NAFLD), which includes its more severe manifestation, non-alcoholic steatohepatitis (NASH), is the most prevalent chronic liver disease worldwide, affecting approximately 25–30% of the global population. The incidence of NAFLD continues to rise annually, correlating with improvements of global living standards (1). NAFLD has emerged as a leading cause of liver cirrhosis, liver transplantation, and hepatocellular carcinoma (HCC). As a metabolic disease, NAFLD is recognized as the hepatic manifestation of metabolic syndrome. Excessive caloric intake, particularly from a high-fat diet (HFD), can lead to NAFLD by promoting chronic inflammation (2). The hallmark of NAFLD is hepatic steatosis, which may occur with or without mild inflammation. As the disease progresses to NASH, the histological features may include lobular inflammation and hepatocyte ballooning, with or without fibrosis (3). During the transition from hepatic steatosis to NASH accompanied by liver fibrosis, the immune system plays an indispensable role (4). In the context of NASH, the liver’s immune cell repertoire is reshaped, contributing to an uncontrolled inflammatory environment that promotes liver injury (hepatocyte death), and fibrosis, thereby exacerbating the disease. Moreover, alterations in liver metabolic functions are now increasingly recognized as the root cause of this disease. Accordingly, NAFLD and NASH have recently been redefined as metabolic dysfunction-associated steatotic liver disease (MASLD) and metabolic dysfunction-associated steatohepatitis (MASH), respectively. However, no drug treatments have been approved for MASLD or MASH to date (5).

JQF is a modified formula developed by Academician Tong Xiaolin derived from the ancient Chinese medical text “Shang Han Lun.” It incorporates the original composition of “Da Huang Huang Lian Xie Xin Tang” and draws on his extensive clinical experience. It has been clinically used with notable efficacy in the treatment of obesity, type 2 diabetes (T2D), and NAFLD, particularly in addressing the “heat state” in T2D patients (6). Moreover, no side effects or adverse reactions have been reported. This conclusion is based on a clinical big data analysis of 4, 561 patients (involving 19, 857 medical visits) diagnosed with T2D or prediabetes and treated at Academician Xiaolin Tong’s clinic from 2000 to 2015 (7). JQF consists of a combination of seven medicinal herbs: *Rheum palmatum (Linné.)* L. (RHEI RADIX ET RHIZOMA), *Coptis chinensis (Linné.)* Franch. (COPTIDIS RHIZOMA), *Anemarrhena asphodeloides (Linné.)* Bge. (ANEMARRHENAE RHIZOMA), *Paeonia lactiflora (Linné.)* Pall. (PAEONIAE RADIX RUBRA), *Citrus reticulata (Linné.)* Blanco (CITRI RETICULATAE PERICARPIUM), *Zingiber officinale (Linné.)* Rosc. (ZINGIBERIS RHIZOMA RECENS), and *Monascus purpureus (Linné.)* Went. (SEMEN ORYZAE CUM MONASCO). In our previous research, we conducted an ingredient analysis and quantitative analysis of JQF reporting that its main components include berberine, epiberberine, coptisine, palmatine, emodin, aloe emodin, timosaponin BII, alibiflorin, paeoniflorin, 6-gingerol, and nobiletin (8). Studies have shown that key components of JQF, including berberine, emodin, timosaponin BII, and paeoniflorin, exhibit notable anti-inflammatory activity and immune regulatory effects. These components can inhibit the release of inflammatory factors such as TNF-α, IL-1β, and IL-6, promote immune responses, alleviate inflammation, and regulate lipid metabolism (9–12). Furthermore, components such as berberine and paeoniflorin have been demonstrated to alleviate hepatic fibrosis (13, 14). Therefore, we speculate that JQF may enhance hepatic lipid metabolism and potentially mitigate hepatic fibrosis in NAFLD and NASH by modulating liver immunity and inflammatory responses.

To comprehensively analyze the cellular and molecular characteristics, as well as the spatial positional information of NAFLD, and the therapeutic mechanisms of JQF intervention in NAFLD, this study combines single-cell RNA sequencing (scRNA-Seq) and spatial transcriptomics (ST). scRNA-Seq enables us to characterize the transcriptomes of individual cells and identify cellular subpopulations within the tissues. On the other hand, ST enables the identification of gene sets expressed in specific cell subpopulations, identified by scRNA-Seq, revealing niches enriched for different gene sets. Integrating scRNA-Seq and ST is essential for linking the pathological phenotypic landscape of tissues to their molecular changes. By integrating data from both techniques, we can gain a deeper understanding of the roles of specific cell subpopulations in NAFLD and their interactions during development, homeostasis, and disease (15, 16). Furthermore, this integration lays a foundation for systematically elucidating the mechanisms of action of JQF in the treatment of HFD-induced NAFLD in mice.

## 2 Materials and methods

### 2.1 Reagents

TNF-α (EMC102aQT.96), IL-1β (EMC001b.96), and IL-6 (EMC004QT.96) elisa kits were purchased from NeoBioscience Technology Co., Ltd (Shenzhen, China). Triglyceride Quantification Colorimetric/Fluorometric Kit (MAK266-1KT) was purchased from Sigma-Aldrich (St. Louis, USA).

### 2.2 Preparation and administration of JQF and metformin

JQF was provided by the pharmacy department of Guang’anmen hospital, China Academy of Chinese Medical Sciences. It consisted of seven medicinal herbs: *Rheum palmatum (Linné.)* L. (RHEI RADIX ET RHIZOMA), *Coptis chinensis (Linné.)* Franch. (COPTIDIS RHIZOMA), *Anemarrhena asphodeloides (Linné.)* Bge. (ANEMARRHENAE RHIZOMA), *Paeonia lactiflora (Linné.)* Pall. (PAEONIAE RADIX RUBRA), *Citrus reticulata (Linné.) Blanco* (CITRI RETICULATAE PERICARPIUM), *Zingiber officinale (Linné.)* Rosc. (ZINGIBERIS RHIZOMA RECENS), *Monascus purpureus (Linné.)* Went. (SEMEN ORYZAE CUM MONASCO) (RHEI RADIX ET RHIZOMA. batch number: 20120030, COPTIDIS RHIZOMA batch number: 21090055, ANEMARRHENAE RHIZOMA batch number: 21100293, PAEONIAE RADIX RUBRA batch number: 21040114, CITRI RETICULATAE PERICARPIUM batch number: 21100120, ZINGIBERIS RHIZOMA RECENS batch number: 21080214, Sichuan New Green Pharmaceutical Technology Development Co., Ltd., Chengdu, Sichuan, China; SEMEN ORYZAE CUM MONASCO batch number: 72030611, Beijing Huamai Pharmaceutical Co., Ltd., Beijing, China). 3g of RHEI RADIX ET RHIZOMA, 15g of COPTIDIS RHIZOMA, 30g of ANEMARRHENAE RHIZOMA, 30g of PAEONIAE RADIX RUBRA, 9g of CITRI RETICULATAE PERICARPIUM, and 6g of ZINGIBERIS RHIZOMA RECENS, were each prepared into granules using conventional methods, and the mixed granules were obtained. Then, 3g of SEMEN ORYZAE CUM MONASCO was decocted in water, filtered, and mixed with the mixed granules. The dosage of JQF is converted according to clinical equivalent doses. In the mouse experiment, the dosage was 14.7875g/kg/d = 9.1 × 1.625g/kg/d (adult standard weight 60kg), which is the equivalent dose for adult clinical use.

Metformin (MET) hydrochloride tablets (batch number: H20023370, Shanghai Sine Promod Pharmaceutical Co., Ltd., China) were provided by the Pharmacy Department of Guang’anmen Hospital, China Academy of Chinese Medical Sciences, diluted in distilled water at a dosage of 300 mg/kg/d (17).

### 2.3 Chemical analysis of JQF

The chemical analysis of JQF was conducted at the preliminary stage of this study using UHPLC-LTQ-Orbitrap HRMS. The major chemical constituents of JQF were identified to be organic acids, alkaloids, and flavonoids, encompassing 63 specific compounds, including malic acid, citric acid, berberine, epiberberine, and nobiletin. Among these, a subset of 10 compounds, including berberine, epiberberine, and emodin, have been recognized as prototype components that manifest in the bloodstream. Additionally, 24 representative components of JQF were subjected to quantitative analysis using UHPLC QqQ MS/MS. For a more comprehensive elaboration of the methodology and findings, please refer to the published article (8).

### 2.4 Experimental animals

Male C57BL/6J mice (22–25g, 5-week-old), were purchased from Gempharmatech Co., Ltd. This animal experiment was conducted in compliance with the ARRIVE guidelines and was strictly performed according to the UK Animals (Scientific Procedures) Act of 1986 and its associated guidelines, the EU Directive for animal experiments 2010/63/EU, and the regulations of the Animal Protection and Use Committee of Guang’anmen Hospital, China Academy of Chinese Medical Sciences, with the ethical approval number (Approval Number: IACUC-GAMH-2022-004). The mice were provided with ad libitum access to water and food and were housed in a specific pathogen-free (SPF) animal facility with controlled temperature (22 ± 2°C) and humidity (40–60%). After 1 week of acclimation, the mice were randomly divided into four groups: control (NCD, n=6), model (HFD, n=7), metformin treatment (MET, n=6), and JQF treatment (JQF, n=6). Except for the NCD group, the remaining mice were induced to develop NAFLD by feeding them a 60% high-fat diet (HFD) (D12492, Research Diets, USA). After 14 weeks of HFD induction, the mice exhibited a significant increase in body weight and blood glucose levels, indicating successful establishment of the NAFLD model. Subsequently, an 8-week intervention was conducted, during which the treatment group was orally administered MET (300 mg/kg/d) and JQF (14.7875 g/kg/d), the NCD group and the HFD group were given distilled water orally, at a dosage of 0.1ml per 10g of animal body weight, once daily. The body weights of the mice were measured weekly, and fasting blood glucose (FBG) levels were measured every 2 weeks. At the end of the intervention, blood samples were collected from the orbital sinus of the mice after anesthesia (1% nembutal, 50mg/kg, IP), and the mice were euthanized using carbon dioxide. In addition, the weight of the liver was measured and the organ-to-body weight ratio was calculated using the formula: (liver weight/body weight) × 100.

### 2.5 Oral glucose tolerance test

After 4 weeks of treatment, all mice were fasted overnight for 14 h and FBG levels were measured. Subsequently, a glucose solution (2 g/kg) was administered by gavage, and blood glucose (BG) levels were monitored through the tail vein at different time points (15, 30, 60, 90, and 120 min). The area under the curve (AUC) values for blood glucose were calculated as follows: AUC=0.5× (BG0min+BG30min)/2 + 0.5× (BG30min+BG60min)/2 + 1× (BG60min+ BG120min)/2.

### 2.6 Biochemical analysis

Serum levels of FBG, alanine aminotransferase (ALT), aspartate aminotransferase (AST), total cholesterol (TC), triglycerides (TG), low-density lipoprotein (LDL), and very low-density lipoprotein (VLDL) were measured using an automatic biochemical analyzer (Baiyang, Beijing). The level of the serum inflammatory factor tumor necrosis factor-alpha (TNF-α), interleukin-1 beta (IL-1β), and interleukin-6 (IL-6) were detected using enzyme-linked immunosorbent assay (ELISA). Liver TG levels were quantitatively measured using a triglyceride assay kit and normalized to the wet tissue weight. The triglyceride-glucose (TyG) index for evaluating the insulin resistance (IR) was calculated as follows: TyG index=ln(TG[mg/dL]× GLU[mg/dL]/2).

### 2.7 Pathological histological analysis

Liver tissue sections embedded in paraffin blocks were subjected to hematoxylin and eosin (H&E) staining, and masson’s trichrome staining (n=3). The overall characteristics of the tissue were observed under a low-power microscope, followed by the observation and representative images were collected from specific regions of interest. Masson’s staining was performed to assess tissue fibrosis, and positive areas were quantified using Aipathwell software.

### 2.8 Immunofluorescence

Liver tissue sections of 3mm thickness were deparaffinized in xylene and subsequently rehydrated through a graded alcohol series (n=3). Microwave antigen retrieval was performed for 10–15 min, followed by cooling to room temperature and blocking with 3% hydrogen peroxide for 30 min at room temperature. The primary antibodies were diluted in 3% bovine serum albumin and incubated overnight at 4°C (using the optimal antibody concentrations determined in pre-experiments: α-SMA 1:100; Colla1 1:1000; Colla2 1:300; F4/80 1:100). Secondary antibodies (enzyme-labeled) were applied at room temperature, followed by the liquid DAB substrate chromogen system. One field of view was randomly selected from each slide and observed at a 100× (100μm) magnification. The sections were imaged under a light microscope. The positive area was quantified using ImageJ software.

### 2.9 Preparation of single-cell suspension

Following hepatic perfusion, mouse liver tissues from the NCD group (n = 1), HFD group (n = 2), and JQF group (n = 1) were collected for scRNA-seq analysis. In brief, the liver samples were cut into small pieces following the manufacturer’s protocol and digested with a digestion solution. A 20uL aliquot of the digestion suspension was stained with Trypan Blue for microscopic examination. The centrifuge tubes were sealed with sealing film and the samples were incubated on a 37°C shaker for 1 h for digestion. After digestion, the suspension was sequentially transferred through 100μm and 40μm filters into centrifuge tubes. The tubes were centrifuged at 300g for 5 min, and the supernatant was discarded. Red blood cell lysis buffer was added to gently disperse cells, followed by a 3-minute incubation. A sufficient amount of wash buffer was added to stop red blood cell lysis. The tubes were centrifuged at 300g for 5 min, and the supernatant was discarded. The cells were resuspended and mixed with the appropriate amount of wash buffer. A 20uL aliquot was taken for AO/PI counting and trypan blue microscopic examination. Based on the quality control results, suitable procedures were selected to remove impurities and dead cells

### 2.10 Preparation and sequencing of single-sell RNA libraries

The chromium instrument and the single cell 3’ reagent kit V3.1 (dual index) was used to prepare individually barcoded single-cell RNA-Seq libraries following the manufacturer’s protocol (10x Genomics). Briefly, sample partitioning and molecular barcoding were done on the chromium controller (10x Genomics), where we loaded cellular suspensions together with the single-cell 3’ gel beads on a single-cell 3’ chip, in which gel beads in emulsion (GEM) generation took place. RNA from the barcoded cells was subsequently reverse-transcribed, and sequencing libraries were constructed using reagents from the chromium single cell reagent kit V3.1 (10x Genomics). Sequencing was performed using Illumina NovaSeq 6000, according to the manufacturer’s instructions.

### 2.11 ScRNA-seq data preprocessing

The raw gene expression matrix was preprocessed using the Cell Ranger pipeline (version 6.0.1) with the mouse reference genome (mm10). Subsequently, the Seurat R package (version 4.0.3) was used for analysis. Following the assessment of varying data quality, low-quality cells were filtered based on sample-specific criteria, and the remaining high-quality cells were subjected to further analysis. The SCTransform function was employed for the normalization and scaling of the samples. High-quality cells were integrated into the matrix and cell clustering was performed based on shared features. The final step involved visualization and analysis of all clustered cells using the uniform manifold approximation and projection (UMAP) algorithm, enabling scRNA-seq visualization and exploration.

### 2.12 Differential gene expression and gene function enrichment analysis

DEG analysis of the cell types in the NCD, HFD, and JQF groups was conducted using the Seurat FindMarkers function. The selection criterion was |avg_log2FC| > 0.25, minimum.pct > 0.1, and adjusted p-value < 0.05. Violin plots or heat maps were generated using the Seurat VlnPlot function and the pheatmap R package (version 1.0.12) for further visualization of the markers. Gene ontology (GO), kyoto encyclopedia of genes and genomes (KEGG), Reactome, and WikiPathways analyses were performed using the clusterProfiler R package (version 3.18.1). These analyses were based on the upregulated and downregulated genes across different groups, with an adjusted p-value < 0.05. The pathway enrichment analysis was performed using the ggplot2 R package (version 3.3.3).

### 2.13 Spatial transcriptomics statistical analysis

We randomly selected mice from the NCD (n = 1) and HFD (n = 1) groups for ST. Space Ranger (version1.3.1) was used for data preprocessing, gene expression quantification and spot identification. A gene spot matrix was generated using Visium spatial barcodes, and then spot clustering and gene expression analyses were performed.

### 2.14 Robust cell type decomposition deconvolution

Based on robust cell type decomposition (RCTD) (18), which is provided by *spacexr* (R package)’s function “*run.RCTD*”, with key parameters set according to default values (eg: doublet_mode set to “full”), we get raw and normalized cell type weight matrix for each cell based count expression matrix from single-cell/spatial transcriptome and cell-type annotation data derived from single-cell transcriptome. The basic principle of RCTD is as follows: first, the average gene expression level of each cell type was calculated in single-cell transcriptome data annotated with cell types. Next, the expression level of each gene in each spot is fitted as a linear combination of the expression levels of each gene across the different cell types. Using the total RNA count of each spot as the input, a statistical model was fitted to estimate the proportions of each cell type in each spot. The cell types identified in spots by RCTD were consistent with those identified in single-cell transcriptomics data.

### 2.15 Reclustering based on cell type abundance

Based on the expression levels of each gene, we performed clustering analysis of spots. However, as each spot may contain cells from multiple cell types, clustering based on gene expression levels may not effectively categorize spots based on their cell-type composition. Clustering analysis of spots based on the abundance ratios of different cell types predicted by RCTD allowed us to determine which spots had similar cell-type compositions. Using a graph-based cluster method (Leiden algorithm in scanpy, resolution = 1) (19), we acquired unsupervised cell cluster result based cell type abundance.

### 2.16 Single-cell regulatory network inference and clustering analysis

To assess transcription factor regulation strength, we applied the single-cell regulatory network inference and clustering (pySCENIC, v0.10.0) (20) workflow using the 20-thousand motifs database for RcisTarget and GRNboost.

### 2.17 Cell communication analysis

To explore the complex network of intercellular communication signals, we conducted an in-depth analysis using the R package CellChat. Based on the default database CellChatDB, we identified signaling pathways, calculated communication probabilities, and constructed communication networks, ultimately determining the relationship between ligand-receptor pair weights and numbers among different cell types, as well as the communication probabilities of ligand-receptor interactions between different cell types. Furthermore, we computed the communication probability at the signaling pathway level by summarizing the communication probabilities of all ligand-receptor interactions associated with each signaling pathway. Each signaling pathway was visualized using Hierarchy plot and Circle plot. In addition, we computed the network centrality scores to identify the signals that contributed the most to the outgoing or incoming signaling of certain cell groups. Subsequently, we inferred the number of patterns using the NMFR package. Finally, we used a River plot to show the associations between latent patterns, cell groups, and signaling pathways.

### 2.18 Statistical analysis

Data were shown as means ± SEM. Multiple comparisons were performed using one-way analysis of variance (ANOVA) and Dunnett’s multiple comparisons test. Student’s t-test was used to compare between two groups. P<0.05 was considered statistically significant.

## 3 Results

### 3.1 JQF significantly improves hepatic steatosis, inflammation, fibrosis and glucose tolerance in NAFLD mice

**Figure 1:**
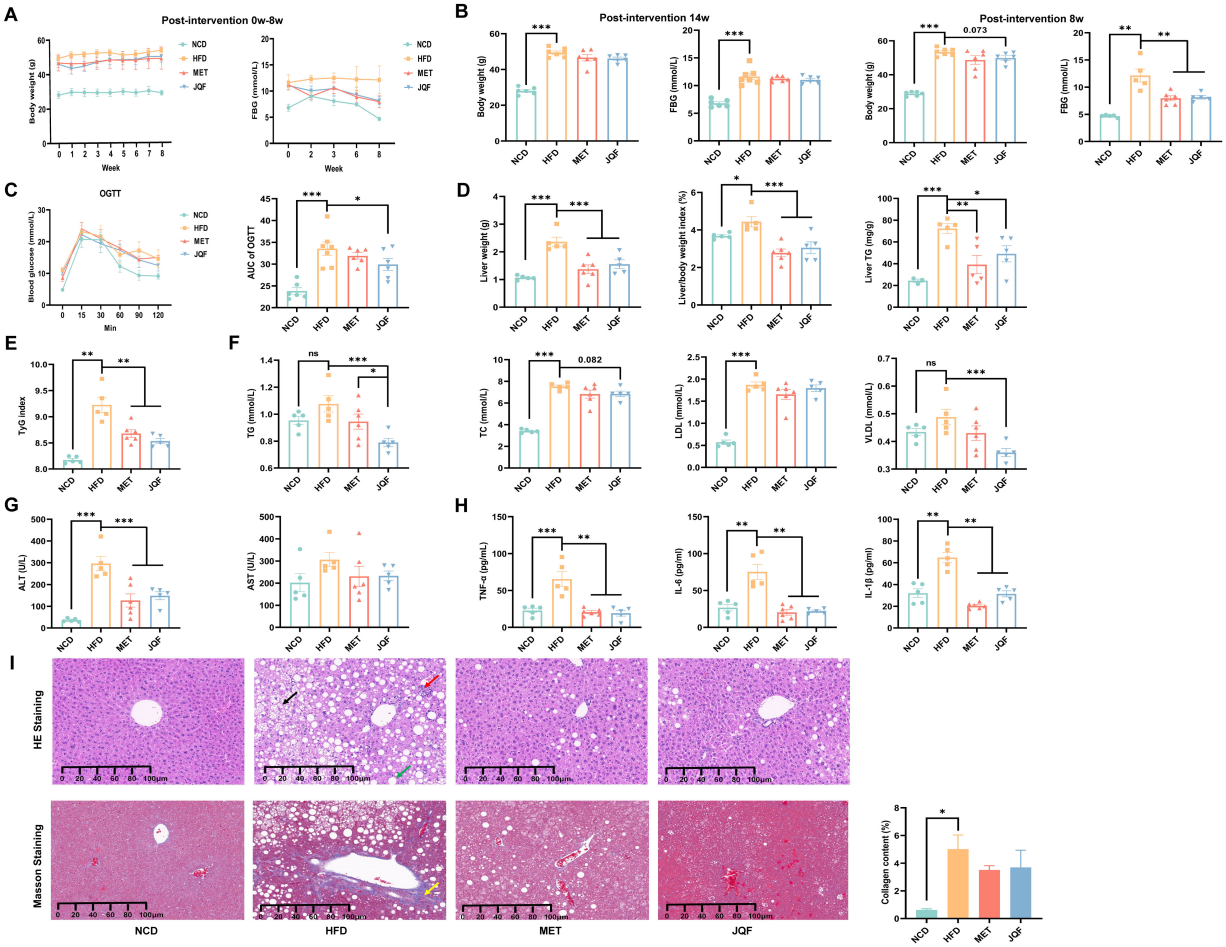
JQF significantly improves hepatic steatosis, inflammation, fibrosis and glucose tolerance in NAFLD mice. **(A)** Effects of the intervention from week 0 to week 8 on body weight and FBG. **(B)** Body weight and FBG at the baseline of successful modeling (i.e., at week 0 of intervention) and before sampling (i.e., after 8 weeks of intervention). **(C)** OGTT and AUC value after 4 weeks of intervention. **(D)** Liver weight, liver index, and hepatic TG. **(E)** TyG index. **(F)** Lipid profile. **(G)** Serum ALT and AST levels. **(H)** Serum levels of inflammatory cytokines TNF-α, IL-1β, and IL-6. **(I)** Hepatic histopathological sections stained with HE, Masson’s trichrome, and collagen fiber scoring, the black arrows indicate the vacuolar changes in hepatocytes with ballooning, the green arrows represent the round vacuoles of steatosis, the red arrows denote lymphocytic and granulocytic infiltration, as well as focal necrosis of hepatocytes, and the yellow arrows signify fibrosis. *P<0.05; **P<0.01; ***P<0.001.

We initiated the establishment of a NAFLD mouse model by inducing it with a HFD for 14 weeks, which was then succeeded by an 8-week therapeutic drug intervention. Compared with the NCD group, the HFD group mice exhibited significant increases in body weight and fasting blood glucose levels. However, JQF was able to significantly suppress these increases in body weight and fasting blood glucose levels (**Figures 1A, B**). After 4 weeks of intervention, OGTT was conducted, which demonstrated that the HFD group mice had significantly reduced glucose tolerance compared with the NCD group. Following glucose administration, blood glucose levels rose rapidly but then declined slowly, resulting in a significant increase in the AUC value. JQF significantly enhanced the glucose tolerance of the mice (**Figure 1C**).

Following 8 weeks of intervention, there was a significant increase in liver weight, liver index, and hepatic TG (**Figure 1D**), a marked alteration in the lipid profile (**Figure 1F**), and notable pathological changes in liver tissue (**Figure 1I**), confirming the hepatic steatosis induced by HFD in mice. Additionally, we observed a significant increase in serum ALT and serum inflammatory factors TNF-α, IL-1β, and IL-6 in the HFD group (**Figures 1G, H**), indicating liver damage and inflammatory responses in NAFLD mice. To further determine the presence of fibrosis in NAFLD mice, Masson’s trichrome staining was performed, revealing a significant increase in collagen fiber content in the HFD group compared with the NCD group, suggesting that NAFLD mice had developed fibrosis and were beginning to transition to the stage of NASH (**Figure 1I**). After intervention with JQF, hepatic steatosis, inflammation, and fibrosis in NAFLD mice were significantly improved. Furthermore, we evaluated hepatic IR via the TyG index (**Figure 1E**), which indicated severe IR in NAFLD mice that was significantly improved by JQF. In summary, JQF not only significantly ameliorated hepatic steatosis, inflammation, and fibrosis in NAFLD mice but also exerted a regulatory effect on glucose metabolism, which may be related to the alleviation of inflammation.

### 3.2 Single cell atlas and spatial characteristics of liver tissue in mice with NAFLD

**Figure 2:**
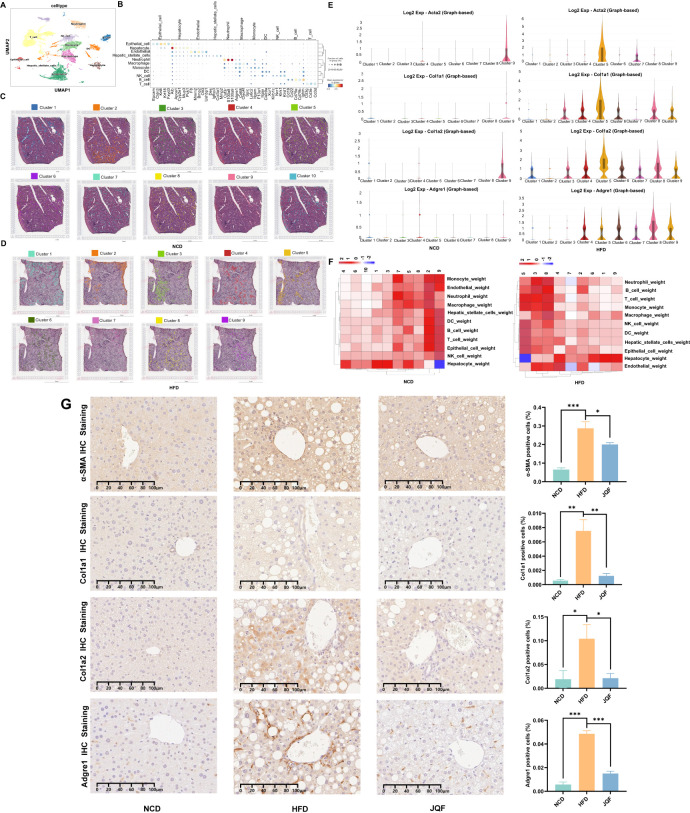
Liver tissue single-cell atlas and spatial features. **(A)** Cellular distribution in the scRNA-seq visualized through UMAP, color-coded by major cell types. **(B)** Dot plot displaying expression of cell type markers for all cell types. **(C, D)** Stepwise cell clustering based on DEGs in the ST, identifying a total of 10 clusters in the NCD group and 9 clusters in the HFD group. **(E)** Expression patterns of fibrosis markers (Acta2, Col1a1, and Col1a2) and macrophage marker (Adgre1) in individual clusters of the NCD and HFD groups within the ST. **(F)** Proportional representation of cell types across different spots, incorporating scRNA-seq data. **(G)** Positive expression and semi-quantitative analysis of fibrosis markers (α-SMA, Col1a1, and Col1a2) and macrophage marker (Adgre1) in the NCD, HFD, and JQF groups. *P<0.05; **P<0.01; ***P<0.001.

To gain a deeper understanding of the cellular and molecular characteristics of NAFLD, we constructed single-cell atlases of NCD and HFD-induced NAFLD mice. After stringent quality control, we obtained a total of 30,474 cells. Dimensionality reduction was performed using principal component analysis (PCA), followed by visualization with the uniform manifold approximation and projection (UMAP) method. A total of 36 cell clusters were identified (**Figure 2A**), which could be categorized into 11 major cell types: epithelial cells (identified by Epcam, Cldn3, Krt8, Fxyd3, and Krt18), hepatocytes (identified by Alb, Apoa1, Cyp2e1, Mup3, and Pck1), endothelial cells (identified by F8, Mmrn2, Bmp2, Oit3, and Ushbp1), hepatic stellate cells (identified by Des, Igfbp3, Col3a1, and Mmp2), neutrophils (identified by S100a8, S100a9, and Csf3r), macrophages (identified by Adgre1, Cd68, Spic, and Clec4f), monocytes (identified by Ly6c2, Itgam, and F13a1), dendritic cells (identified by Itgax, Xcr1, and Clec9a), NK cells (identified by Klrb1c, Ncr1, Klrk1, and Klrd1), B cells (identified by Cd19, Cd22, Cd79a, Cd79b, and Ighm), and T cells (identified by Cd3g, Cd3e, and Cd3d) (**Figure 2B**).

Furthermore, we performed ST on the liver tissues of NCD and NAFLD mice to gain insights into the spatial localization of different cell types within the liver. Based on the expression levels of each gene, we conducted a clustering analysis of the spots. The NCD group had 10 identified clusters, while the HFD group had 9 identified clusters (**Figure 2C**). However, since each spot may contain cells from multiple cell types, clustering based solely on gene expression levels might not effectively categorize the spots according to their cellular composition. Therefore, we relied more heavily on scRNA-seq data and employed deconvolution analysis based on RCTD. This approach yielded raw and normalized cell type weight matrices derived from single-cell/spatial transcriptomics, as well as cell type annotation data extracted from single-cell transcriptomics. Furthermore, the clustering analysis of spots based on the predicted abundance ratios of different cell types by RCTD allowed us to identify spots with similar cellular compositions. Combining this information with our scRNA-seq data, we obtained a proportion plot showing the distribution of cell types in different spots (**Figure 2D**), such as Guo (21). Interestingly, we found that the endothelial cells in NAFLD mice were surrounded by various inflammatory and immune cells, including neutrophils, monocytes, B cells, macrophages, and T cells. Additionally, the number of hepatocytes in this area was significantly reduced, which was not observed in NCD mice. Moreover, we found that this region exhibited the most severe inflammation and fibrosis, particularly in the areas infiltrated by T cells. The macrophage marker F4/80 (Adgre1) and fibrosis markers (α-SMA, Col1a1, and Col1a2) were highly expressed in this region (**Figure 2E**). Immunohistochemical staining confirmed the presence of inflammation and fibrosis in the livers of NAFLD mice, which were significantly improved by JQF treatment (**Figure 2F**).

### 3.3 JQF alleviates NAFLD by modulating the immune cell population

**Figure 3:**
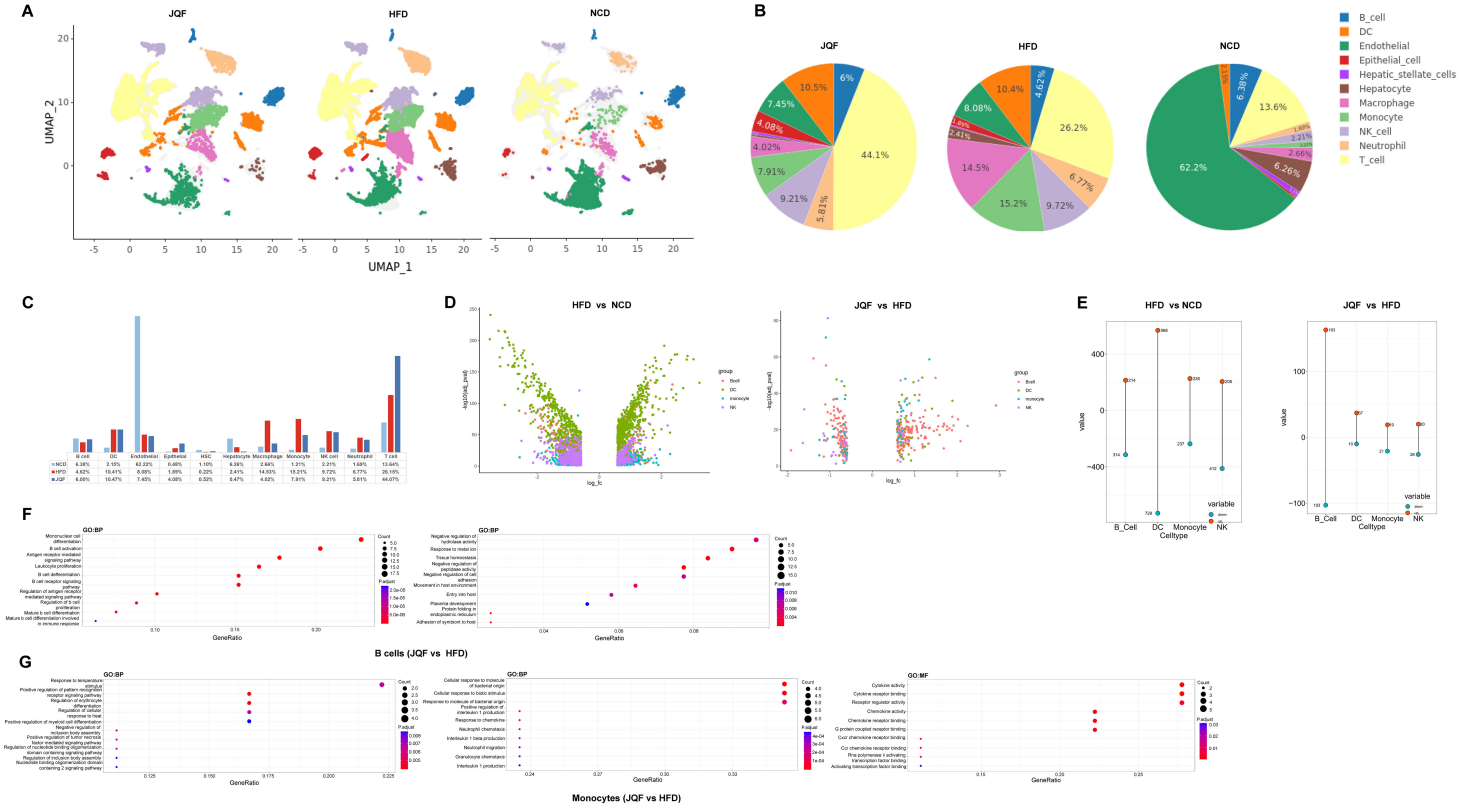
JQF improves NAFLD by modulating immune cell populations. **(A)** Single-cell atlas for each group. **(B, C)** Proportions of cell types in each group. **(D, E)** Differential expression analysis of B cells, DCs, monocytes, and NK cells within cell clusters, volcano plots showing DEGs, and the number of upregulated and downregulated DEGs. The upregulated genes are represented in red, and downregulated genes are represented in green. **(F, G)** GO enrichment analysis (BP, biological processes),(MF, molecular functions) for B cells and monocytes, showing the top 10 enriched terms.

We further conducted independent analyses of the NCD, HFD, and JQF groups on the basis of the constructed single-cell atlas, characterizing the single-cell landscapes of each group (**Figure 3A**). We performed independent analyses of the cell proportions for each cell type in these three groups and identified changes in the proportions of multiple cell types between the HFD and NCD groups (**Figures 3B, C**). Additionally, we found that JQF improved some of these differences. For example, the percentages of macrophages and monocytes were significantly greater in the HFD group than in the NCD group but significantly lower in the JQF group. Furthermore, compared with those in the NCD group, the percentages of T cells, natural killer (NK) cells, dendritic cells (DCs), and neutrophils were significantly greater in both the HFD and JQF groups, whereas the percentages of hepatocytes and endothelial cells were significantly lower. Notably, the percentage of T cells showed significantly changed after JQF treatment. These findings indicate significant alterations in immune cell populations in the liver tissue of HFD-induced NAFLD mice, and that JQF can modulate some of these immune cells.

To gain a better understanding of the functions and roles of each immune cell population in this study, and to elucidate the roles of innate and adaptive immune response cells in NAFLD, we performed differential expression analysis within and between cell clusters for B cells, DCs, monocytes, and NK cells (**Figure 3D**). We found that, compared with the NCD group, DCs had the greatest number of differentially expressed genes (DEGs) in the HFD group, totaling 1297 DEGs. NK cells, B cells, and monocytes were also affected, with 617, 527, and 463 DEGs, respectively. Interestingly, after JQF treatment, B cells became the cell population with the greatest number of regulated DEGs, totaling 266. Although there was no significant change in the proportion of B cells among the three groups, the numbers of DEGs regulated by DCs, NK cells, and monocytes were only 47, 46, and 40, respectively (**Figure 3E**).

Next, we conducted enrichment analysis on the 266 DEGs that showed the most significant changes in B cells after treatment. The GO terms revealed involvement in the B cell receptor signaling pathway, B cell activation, differentiation of mature B cells in immune response, regulation of antigen receptor-mediated signaling pathway, and regulation of B cell proliferation (**Figure 3F**). These findings suggest that B cells can secrete cytokines and antibodies, regulate immunity, promote inflammation, and also negatively regulate the activity of hydrolases, participate in protein folding within the endoplasmic reticulum. Endoplasmic reticulum stress can lead to abnormal expression and function of endoplasmic reticulum-related proteins, contributing to lipid metabolism disorders and exacerbating NAFLD (22). Additionally, we performed enrichment analysis on the DEGs of monocytes, which showed a significant decrease in cell proportion after treatment. We found that monocytes can positively regulate tumor necrosis factor-mediated signaling pathway and interleukin-1 production, and can interact with Cxcr chemokine receptors and Ccr chemokine receptors, thus mediating cytokine activity (**Figure 3G**). These findings indicate that monocytes play a role in regulating inflammatory responses in NAFLD through the release of cytokines and chemokines. Moreover, monocytes can be activated and recruited to liver tissue, where they further differentiate into macrophages, and promote liver inflammation and fibrosis (23). These findings suggest that JQF may regulate inflammation and immune dysregulation in NAFLD.

### 3.4 The identification of macrophage subclusters revealed the crucial role of innate immunity in NAFLD

**Figure 4:**
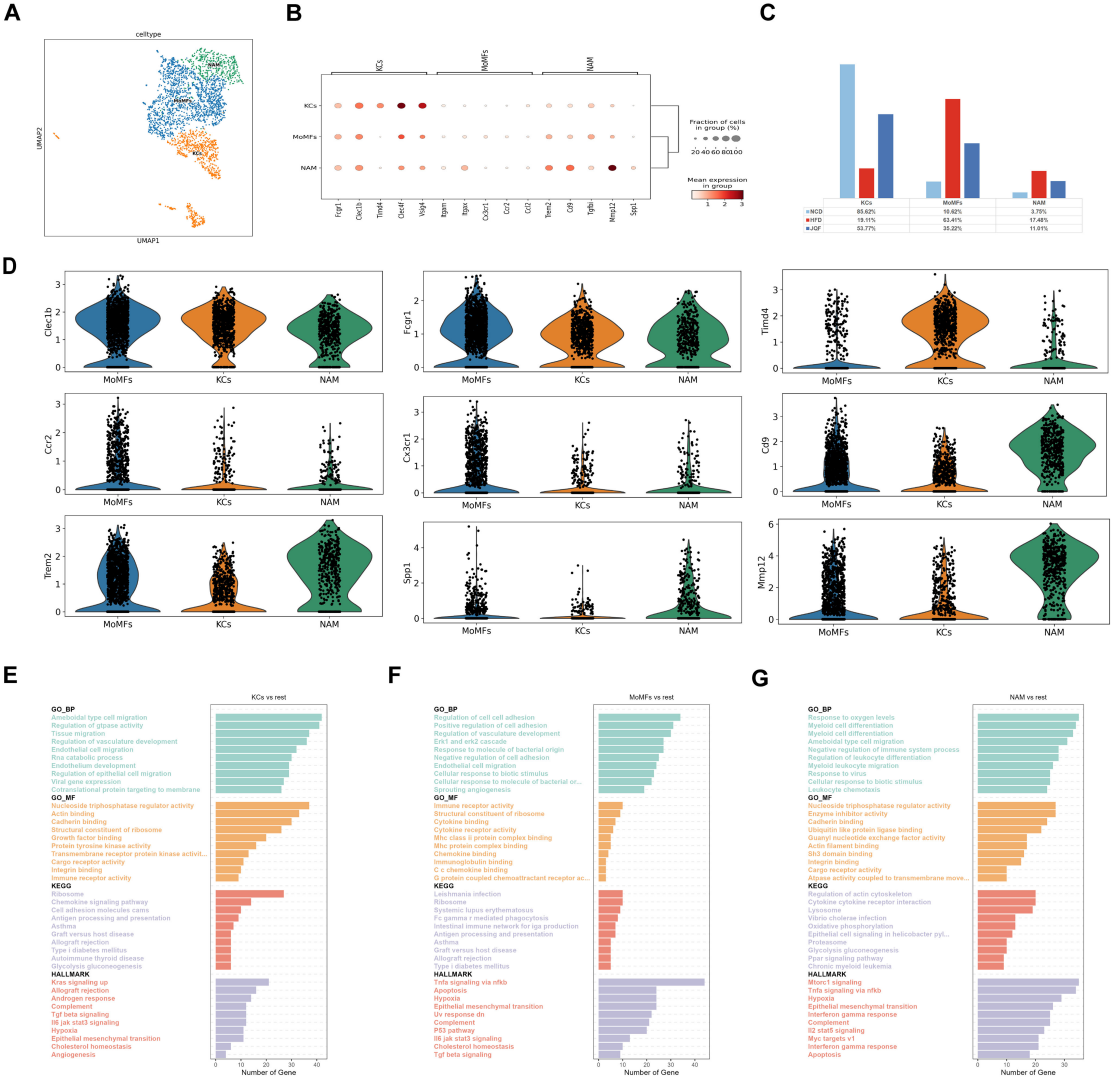
Identification of macrophage subclusters. **(A)** Subclustering of macrophages was performed using UMAP visualization. **(B)** Dot plot was used to visualize the expression of cell type markers in the macrophage subclusters. **(C)** Proportions of cell types within each group of macrophage subclusters. **(D)** Circos plot illustrating the prominent molecules in the macrophage subclusters. **(E–G)** Top 10 enriched terms in GO (BP, MF), HALLMARK, and KEGG pathway analysis for the macrophage subclusters.

Liver macrophages exhibit heterogeneity. To comprehensively characterize the changes in macrophages in NAFLD and their response to JQF intervention, we identified three subgroups of macrophages (**Figures 4A, B**). We further compared the proportions of different macrophage subgroups among the three groups (**Figure 4C**). In the HFD group, the percentage of monocyte-derived macrophages (MoMFs) was significantly greater than that in the NCD group, while the proportion of tissue-resident Kupffer cells (KCs) was significantly lower. Normally, KCs are the resident macrophages in the liver, accounting for 80–90% of all macrophages. However, as NAFLD progresses to NASH, the number of KCs gradually decreases, and MoMFs infiltrate and increase, replacing the resident KCs as the predominant macrophage population (24). Our results are consistent with these findings. After JQF intervention, the proportions of MoMFs and KCs significantly changed, with a trend toward the NCD group. Additionally, we identified a macrophage subgroup that expresses lipid receptors, known as NASH-associated macrophages (NAM), which express triggering receptors on myeloid cells 2 (TREM2) and expand in NASH livers to coordinate hepatocyte energy supply and mitochondrial function (25). We found that the proportion of NAM was significantly greater in the HFD group than in the NCD group, and that it decreased after JQF intervention. These findings suggest that JQF treatment significantly alters the proportions of macrophage subgroups, indicating that JQF may primarily exert its therapeutic effects by regulating KCs and MoMFs. Upon further analysis of the subpopulations, we found that the prototypic marker Clec1b was highly expressed in KCs, as was the characteristic expression of Timd4 (**Figure 4D**). The enrichment analysis revealed that KCs are enriched in processes related to lipid engulfment through endocytosis, regulation of cholesterol homeostasis, as well as pathways associated with inflammation and fibrosis, such as chemokine signaling, angiogenesis, and epithelial-to-mesenchymal transition. Additionally, KCs are enriched in processes related to antigen processing and presentation, indicating their involvement as antigen-presenting cells in antigen processing and presentation (**Figure 4E**). MoMFs exhibited high expression of the pan-macrophage marker Fcgr1, as well as Clec1b. Monocyte infiltration was dependent on chemokine receptors such as Ccr2 and Cx3cr1 (**Figure 4D**). Enrichment analysis indicated that MoMFs were engaged in phagocytosis mediated by Fcγ receptors, transduction of Tnfa signal transduction through nfkb, Il6 jak stat3 signaling promoting inflammation, and immune regulation through association with major histocompatibility complex (MHC) II protein complexes. Furthermore, MoMFs were enriched in fibrosis-related processes such as sprouting angiogenesis, positive regulation of cell adhesion, regulation of vascular system development, and TGF-β signaling pathway (**Figure 4F**). Moreover, NAMs exhibited characteristic expression of Cd9, Trem2, and Spp1 (**Figure 4D**). Spp1, encoding osteopontin (OPN), is a potent inducer of liver fibrosis. NAMs also highly expressed genes involved in tissue repair and remodeling, such as Mmp12, and were consistently enriched in GO terms related to lipid metabolism, collagen synthesis, and tissue remodeling, including Ppar signaling pathway and regulation of actin cytoskeleton (**Figure 4G**).

### 3.5 The identification of T cell subclusters revealed the crucial role of adaptive immunity in NAFLD

**Figure 5:**
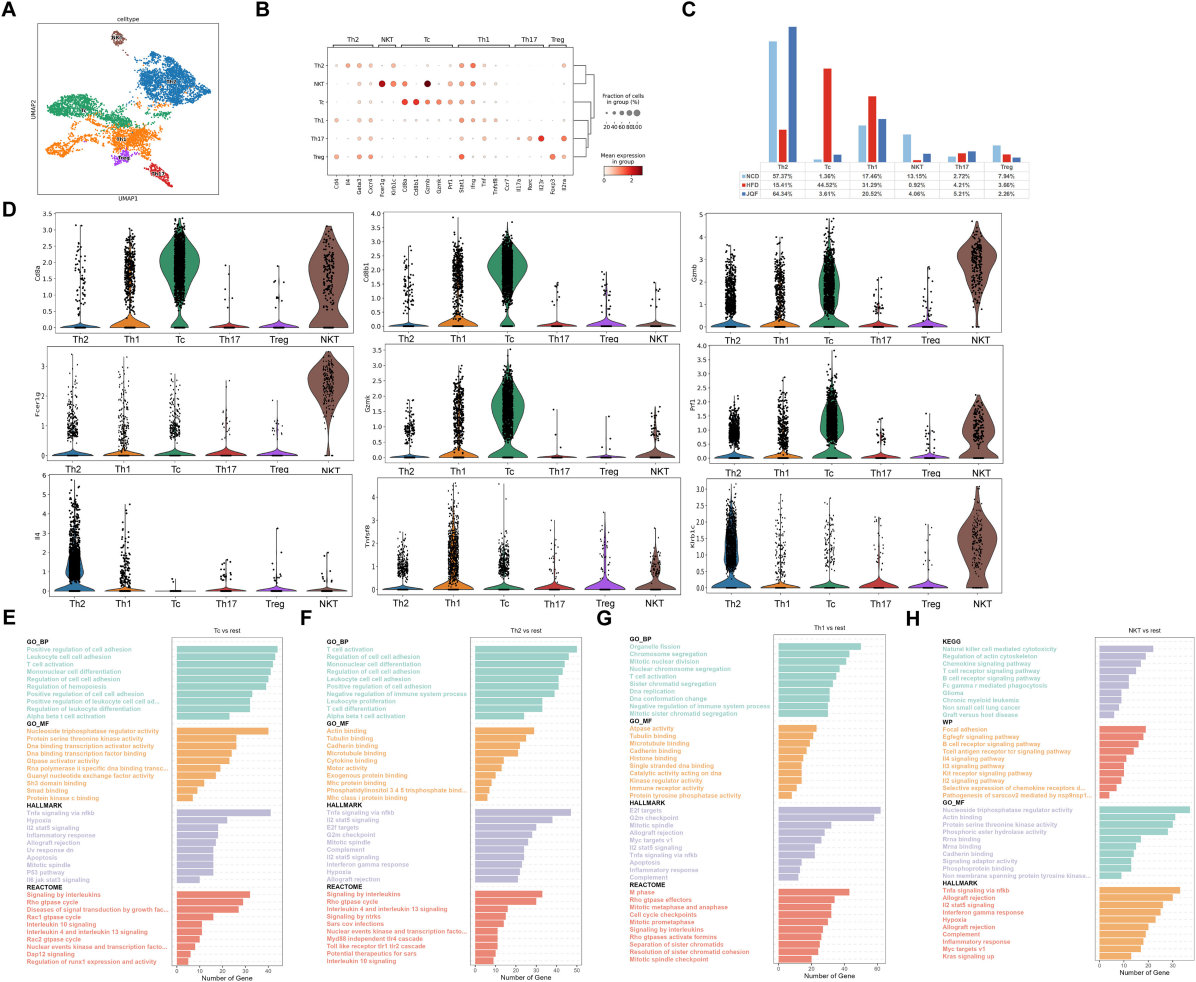
Identification of T cells subclusters. **(A)** Subclustering of T cells was performed using UMAP visualization. **(B)** Dot plot was used to visualize the expression of cell type markers in the T cells subclusters. **(C)** Proportions of cell types within each group of T cells subclusters. **(D)** Circos plot illustrating the prominent molecules in the T cells subclusters. **(E–H)** Top 10 enriched terms in GO (BP, MF), REACTOME, HALLMARK, WP, and KEGG pathway analysis for the T cells subclusters.

We also performed subclustering of T cells and identified six T cell subgroups, including Th1, Th2, Tc, NKT, Treg, and Th17 (**Figures 5A, B**). We compared the proportions of these subgroups among the three groups and found that the percentages of Th2 and NKT cells were significantly lower in the HFD group than in the NCD and JQF groups. In contrast, the percentages of Tc and Th1 cells were significantly greater in the HFD group than in the NCD group. After JQF intervention, the proportions of these cell subgroups significantly decreased (**Figure 5C**). These findings indicate that JQF treatment leads to significant changes in the proportions of T cell subgroups, especially Th2 and Tc subgroups. Further analysis revealed that the Tc subgroup, which showed significant changes in cell proportions, exhibited characteristic expression of Cd8a, Cd8b1, Gzmb, Gzmk, and Prf1 (**Figure 5D**). Studies have shown that lobular inflammation and ballooning are associated with the accumulation of Tc cells in the liver (26). Tc cells may participate in inflammatory responses and cytotoxicity by recognizing and killing damaged liver cells or by releasing cytotoxic molecules (such as perforin) to induce apoptosis of damaged cells, thus contributing to liver inflammation and fibrosis. GO enrichment analysis also identified consistent terms related to cell killing, positive regulation of cell adhesion, regulation of immune effector process, T cell activation, inflammation, and cell apoptosis (**Figure 5E**). This suggests that Tc cells may be a key cell population involved in the alleviation of NAFLD by JQF. Another subgroup showing significant changes in cell proportions was Th2, which exhibited characteristic expression of Il-4 (**Figure 5D**). DEG analysis revealed enrichment of biological processes related to interleukin-4 and interleukin-13 signaling, as well as interleukin-10 signaling. Th2 cells can also interact with MHC class I proteins to regulate T cell activation and negatively regulate immune system processes (**Figure 5F**). This may be another reason for the therapeutic effects of JQF. Furthermore, Th1 cells showed characteristic expression of Tnfsf8 and Mki67 (**Figure 5D**). The main effector functions of Th1 cells involve cellular immunity and inflammation, including the activation of other immune cells such as macrophages and natural killer cells. Enrichment analysis also suggested that Th1 cells can participate in natural killer cell-mediated immune regulation and regulate T cell activation (**Figure 5G**). NKT cells exhibited characteristic expression of Fcer1g, Klrb1c (encoding NK1.1), and Gzmb, as well as Cd8a (**Figure 5D**). NKT cells are considered to be a subset of T cells that express the T cell receptor (TCR) and Fcer1g with high cytotoxic potential. KEGG analysis also revealed their involvement in natural killer cell-mediated cytotoxicity and T cell receptor signaling pathways (**Figure 5H**).

### 3.6 Immune subgroups mitigate NAFLD by exerting anti-inflammatory and anti-fibrotic effects through cellular communication

**Figure 6:**
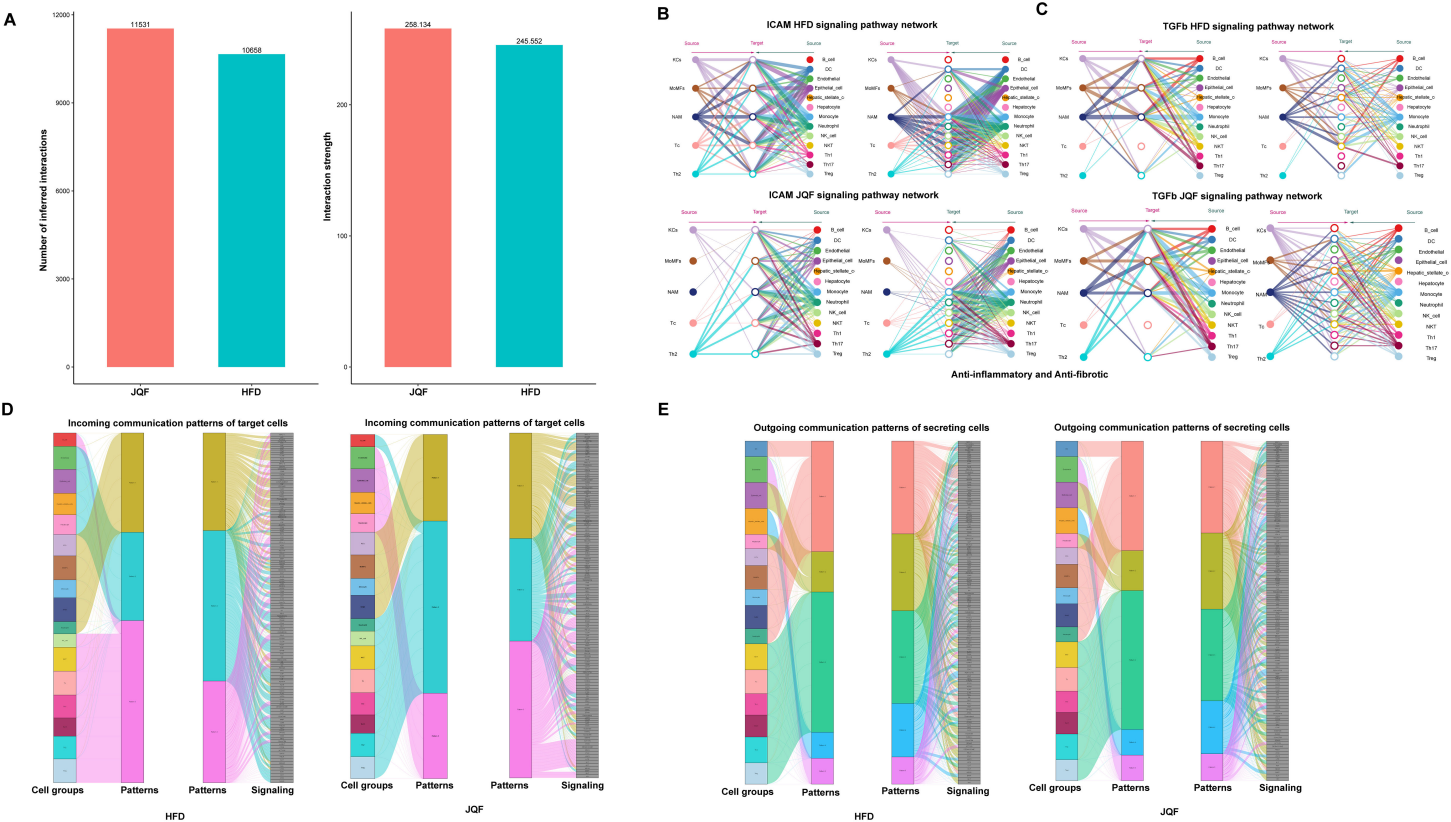
Cell communication analysis after JQF treatment for NAFLD. **(A)** Number of inferred interactions and interaction strength. **(B)** Hierarchical diagram of the inferred cellular communication network for ICAM signaling. **(C)** Hierarchical diagram of the inferred cellular communication network for TGFb signaling. **(D)** Inferred incoming communication patterns for target cells. **(E)** Inferred outgoing communication patterns for secretory cells, showing the correspondence between inferred potential patterns, cell groups, and signaling pathways.

Macrophage subgroups KCs, MoMFs, NAM, and T cell subgroups Th2 and Tc play significant roles in NAFLD, and they may be the key subgroups through which JQF exerts its therapeutic effects. To further clarify how these immune subgroups contribute to treatment, we conducted a cell communication analysis on 18 cell populations (including subgroups) divided into HFD and JQF groups. The results revealed that overall cell communication was enhanced after JQF treatment, including an increase in the number of inferred interactions and increased interaction strength, was increased after JQF treatment (**Figure 6A**). We focused on two representative signaling pathways within the significant ligand-receptor pairs, including the ICAM and TGFb pathways. The inferred ICAM signaling network indicated that DC cells, epithelial cells, monocytes, and neutrophils are the most prominent sources of ICAM ligands that act on macrophages (KCs, MoMFs, NAM) and T cells (Th2 and Tc). After JQF treatment, communication targeting macrophages was significantly reduced, substantially weakening both autocrine and paracrine signaling in macrophages while enhancing autocrine and paracrine signaling in Th2 cells, thereby reducing inflammation and alleviating fibrosis. This finding is consistent with that of Chen et al., who reported that weakened ICAM signals and reduced intercellular adhesion molecule crosstalk between activated hepatic stellate cells and neutrophils mitigated liver fibrosis (27) (**Figure 6B**). Additionally, the inferred TGFb signaling network suggested that monocytes and B cells are the main sources of TGFβ ligands that act on macrophages. Studies also indicate that TGF-β is a critical regulator of both innate and adaptive immunity, acting as a general executor of immune tolerance and an inflammation suppressor. Lack of TGF-β can lead to inflammation, thereby causing fibrosis. TGF-β can inhibit B cell proliferation and significantly affects macrophage signal transduction, *In vitro*, culturing tissue macrophages with TGF-β can suppress the expression of various pro-inflammatory genes, including TNF, IL-12, and inducible nitric oxide synthase (28). After JQF treatment, the communication of genes involved in the TGFb signaling pathway, especially Th2 cells, was increased. Notably, CellChat revealed that most of the TGFb interactions between these cells were paracrine with only macrophages (KCs, NAM), monocytes, and B cells exhibiting significant autocrine signals (**Figure 6C**).

In addition to exploring the detailed communication of individual pathways, we also employed a pattern recognition approach based on non-negative matrix factorization to identify global communication patterns and key signals within different cell populations, exploring how multiple cell groups and signaling pathways coordinate their effects (29). This analysis revealed three incoming signal patterns and five outgoing signal patterns. For example, the communication patterns of target cells indicate that incoming signals to macrophages and T cells were predominantly governed by patterns #1 and #3, which include pathways such as TGFβ and ICAM, and signals like CD86, TNF, CSF, and SPP1. Furthermore, JQF treatment could alter the communication patterns of cells, such as incoming B cell signals in the HFD group, which were predominantly governed by pattern #3 before treatment, shifting to pattern #2 following JQF treatment (**Figure 6D**). On the other hand, the output revealed that all outgoing T cell signals were characterized by pattern #3, representing various pathways including but not limited to MHC-1, CCL, CD6, IFN-II, and IL4. It also showed that all outgoing macrophage signals were characterized by pattern #1, which represented pathways such as TGFβ, TNF, IL1, and PDGF (**Figure 6E**). Additionally, it is noteworthy that both the incoming and outgoing signals of monocytes shared the same pattern #1 with macrophages. Overall, this suggests that despite the differences in cell types, shared signaling networks are possible within the same tissue, and JQF treatment can alter the communication patterns of certain cells.

### 3.7 SCENIC analysis revealed key transcription factors regulating the progression of NAFLD

**Figure 7:**
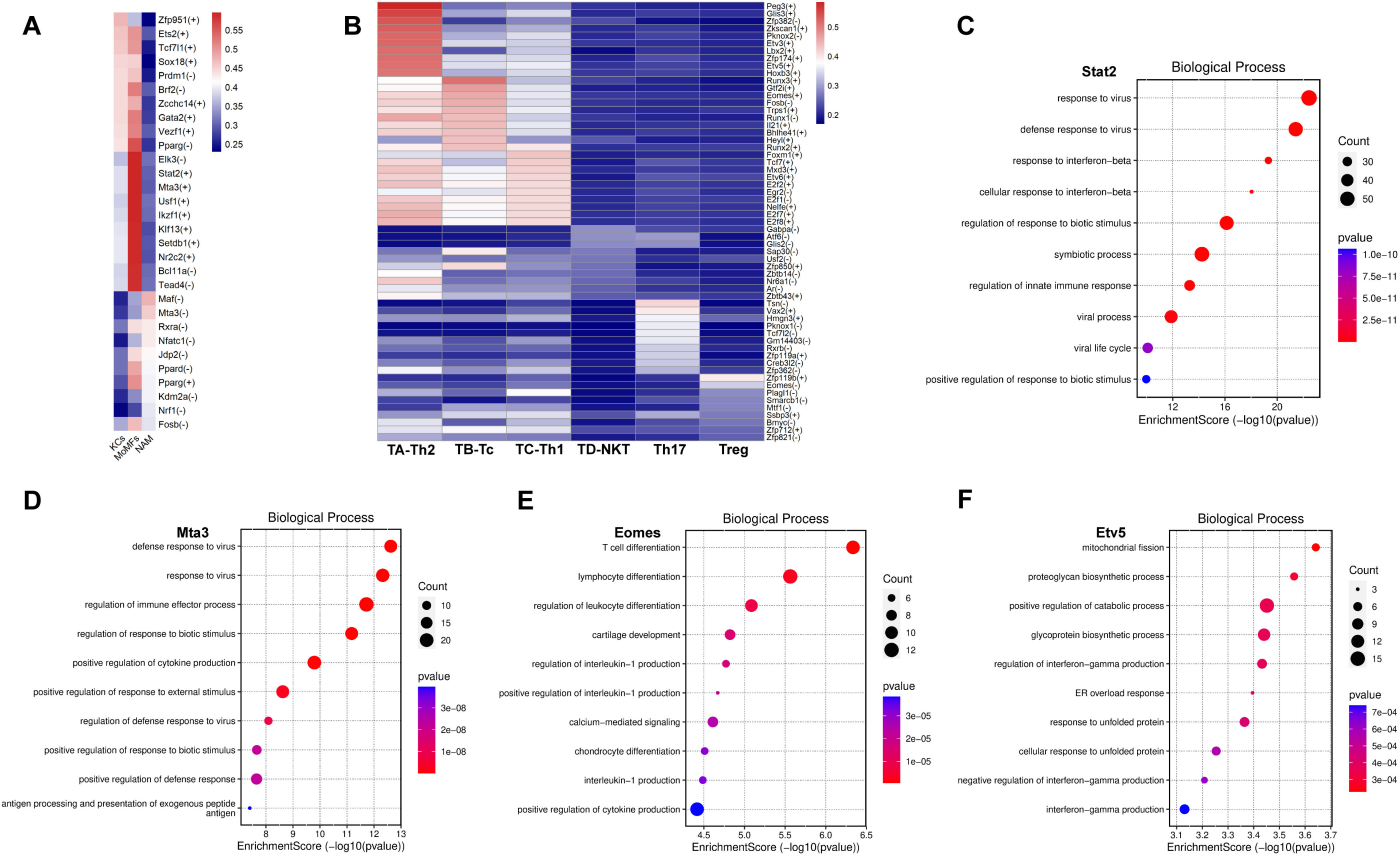
Key TFs regulated by JQF in NAFLD. **(A, B)** Top 10 key TFs identified for subclusters of macrophages and T cells based on RSS values. **(C–F)** Top 10 enriched GO terms in BP for TFs Stat2, Mta3, Eomes, and Etv5.

To further explore the gene regulatory networks governing the homeostasis and activation of macrophages and T cells, we performed unbiased single-cell regulatory network inference and clustering (SCENIC) analysis on their respective transcriptomes. In macrophages, we identified 528 direct homologous transcription factors and created regulon specificity scores (RSS) based on Jensen-Shannon divergence. We visualized the top 10 key transcription factors with the highest RSS values in each macrophage subgroup (**Figure 7A**). Additionally, we found that Stat2, an important transcription factor (TF) in the JQF-regulated macrophage subgroup called MoMFs, may be a critical transcription factor involved in JQF-mediated alleviation of NAFLD inflammation. Enrichment analysis revealed its role in regulating innate immune responses and playing a crucial role in cellular signaling of interferons (**Figure 7C**). Studies have shown that defects in Stat2 can impair type I interferon signaling, leading to excessive inflammation (30). Another significant TF in MoMFs, Mta3, was found to positively regulate cytokine production and play a crucial role in antigen processing and presentation of exogenous peptide antigens, thereby regulating immune effector processes (**Figure 7D**). Furthermore, our results indicated that the Usf1 TF in MoMFs and the Pparg TF in KCs are key regulators of hepatic lipid metabolism (**Figure 7A**). Studies have shown that in mice, inactivation of Usf1 significantly improves diet-induced dyslipidemia, obesity, IR, hepatic steatosis, and atherosclerosis (31), while NASH is associated with IR and increased expression of PPARγ in the liver (32).

In T cells, we identified 564 direct homologous transcription factors and created an relative signal strength (RSS) heatmap to visualize the top 10 key transcription factors with the highest RSS values in each T cell subgroup (**Figure 7B**). Furthermore, we conducted enrichment analysis on the significantly altered cell subgroups Tc and Th2 after JQF treatment. The results indicated that the Eomes transcription factor in the Tc subgroup can regulate leukocyte differentiation and participate in lymphocyte and T cell differentiation (**Figure 7E**). Eomes positively regulates the production of cytokines, including interleukin-1. Eomes has been reported as a major transcription factor in Tc cells, closely associated with CD8 T cell development and effector functions (33). Therefore, we consider Eomes a potential key transcription factor in JQF-mediated immune modulation for alleviating NAFLD in Tc cells. Another key cell subgroup significantly regulated by JQF, Th2, was enriched with the Etv5 transcription factor. Enrichment analysis revealed that Etv5 actively regulates processes such as protein degradation and glycoprotein biosynthesis, responds to endoplasmic reticulum stress overload, and participates in cellular responses to unfolded proteins (**Figure 7F**). Research has demonstrated that Etv5 is an obesity-related transcriptional inhibitor of insulin secretion and is closely associated with obesity and type 2 diabetes (34). Mao Z et al. demonstrated that peroxisome proliferator-activated receptor (PPAR) signaling and fatty acid degradation/metabolism pathways are significantly downregulated in Etv5-deficient hepatocytes both *in vitro* and *in vivo*. Etv5 was identified as a novel transcription factor regulating hepatic fatty acid metabolism (35). Additionally, Etv5 negatively regulates the production of interferon-gamma. Therefore, we consider Etv5 as another important transcription factor in JQF’s therapeutic effects.

## 4 Discussion

NAFLD is a common metabolic disorder that has received significant attention due to its increasing prevalence and its potential to progress to liver dysfunction and failure. It has recently been renamed as MASLD. Currently, there are no approved treatments for NAFLD (36). JQF is a formula developed by Academician Tong Xiaolin based on decades of clinical experience. It is derived from the modified classic formula Da Huang Huang Lian Xiexin Tang and has shown promising therapeutic effects on obesity, NAFLD, and T2D. NAFLD progression features include simple lipid accumulation, NASH, steatosis, inflammation, hepatocyte injury, and fibrosis. The liver, an important immune organ, contains numerous innate and adaptive immune cells. Studies have demonstrated that NAFLD involves several immune cell-mediated inflammatory processes, particularly during the progression to NASH, where inflammation is a key component of the disease (22, 37). The emergence of scRNA-seq and ST has provided more precise tools to comprehensively reveal the characteristics and spatial information of liver immune cells during NAFLD and NASH, and to elucidate the mechanisms underlying the therapeutic effects of JQF.

Firstly, JQF significantly improved the liver weight, liver index, hepatic TG, and lipid profile in NAFLD mice, and it also significantly decreased serum ALT and inflammatory levels, alleviating the histopathological changes in the liver. These findings confirm that JQF improves hepatic steatosis, inflammation, and fibrosis in NAFLD mice. Additionally, JQF was able to significantly inhibit weight gain and blood glucose elevation in NAFLD mice, enhance the body’s glucose tolerance, and alleviate IR. Besides, JQF did not exhibit any adverse reactions or side effects in the treatment of NAFLD mice. By combining scRNA-seq and ST, we performed transcriptional mapping of all major resident cell types in NAFLD. We obtained spatial positional information and further explored the potential mechanisms of JQF at the single-cell level. In this study, we constructed a single-cell atlas of liver tissue and identified 11 major cell types along with their gene expression profiles. We also observed distinct immune cell distributions between NCD mice and NAFLD mice, with the onset of fibrotic phenotypes in the immune cell-infiltrated areas of NAFLD mice, indicating the potential roles of immune cells such as monocytes, macrophages, and T cells in the progression of NAFLD, consistent with other research reports (22). Taken together, our scRNA-seq data suggest that JQF significantly modulates these immune cell populations. Cellular communication analysis also confirmed the relationships among macrophages, monocytes, and B cells.

Further investigation revealed that monocytes, macrophage subtypes KCs and MoMFs, and T cell subtypes Th2 and Tc were the most immune-regulated cells in the liver tissue of NAFLD mice treated with JQF. Liver macrophages, namely KCs and MoMFs, are reported to be key participants in innate immunity and are highly plastic within the immune system. Compared with KCs, MoMFs are more pro-inflammatory and influence the liver’s response to NASH by limiting lipid storage and promoting liver injury (37). The activation of the immune system and the recruitment of pro-inflammatory cells to the liver during the NASH stage are critical events in the pathogenesis of NAFLD, with hepatic macrophages having been shown to promote the progression of NAFLD by producing inflammatory factors (38). Consistent with our findings, JQF increased the proportion of KCs, decreased the proportions of MoMFs and NAMs, regulated macrophage remodeling, alleviated inflammatory responses, and participated in immune regulation through MHC II antigen presentation. The transcription factor Stat2 in the MoMFs subset may play a key upstream role in JQF’s effects. Studies have shown that Stat2, as a member of the Stat family, plays a crucial role in immune responses to both extracellular and intracellular stimuli, including cancer initiation, inflammatory responses, and tumor cell invasion. Loss of Stat2 may lead to aberrant inflammatory signaling transduction in macrophages. Additionally, Stat2 is involved in lipid synthesis by regulating acetyl-CoA carboxylase 1 (ACC1) levels (39, 40), indicating that Stat2 may be a key transcription factor in the MoMFs subset involved in the alleviation of hepatic steatosis and inflammation in NAFLD by JQF. Furthermore, our results suggest that the transcription factor Mta3 in the MoMFs subset may be a key factor in the ability of JQF to alleviate hepatic fibrosis in NAFLD. It has been reported that Mta3 participates in fibrosis by inhibiting epithelial-mesenchymal transition (EMT). A study by Xiao et al. showed that emodin, one of the key components of JQF, alleviated cardiac fibrosis by upregulating Mta3 to inhibit the activation of cardiac fibroblasts (41). Therefore, emodin may contribute to JQF’s effects.

Our research suggests that JQF primarily ameliorates NAFLD by modulating Th2 and Tc cells involved in adaptive immunity. T cells induce specific effector functions by recognizing antigens presented on MHC class I molecules by antigen-presenting cells (42). It has been reported that the activation of Tc cells enhances the secretion of pro-inflammatory cytokines, such as IFNγ and TNFα, and chemokines crucial for macrophage recruitment and adipose tissue inflammation, playing a pivotal role in the progression of NASH (43). In both mice and humans with NASH, there is an increase in liver CD8 (+) T cells, which predominantly produce cytotoxic molecules such as IFNγ, TNF, and perforin. Depleting CD8 (+) T cells can restore liver insulin sensitivity, reduce liver injury, and decrease fibrosis. These CD8 (+) T cells are characterized by the expression of activation and exhaustion markers and the immune checkpoint molecule programmed cell death protein 1 (PD-1), indicating their potential to promote liver injury during NASH (37, 44). Our results indicate that JQF significantly reduces the proportion of Tc cells, and the DEGs also suggest that Tc cells positively regulate cell killing, cell adhesion, T cell activation, inflammation, and cell apoptosis, which play important roles in NAFLD and NASH progression. Through SCENIC analysis, we predicted the upstream transcription factor Eomes, a key participant in the effector functions and differentiation of CD8 (+) cytotoxic T lymphocytes (45). Conversely, Th cells assist macrophages, effector T cells, and B cells in eliminating pathogens and infected cells. In NAFLD, Th2 cells that produce IL-4 and/or IL-13 seem to exert anti-inflammatory effects, but the immune mechanisms mediated by Th2 cells in NAFLD and NASH are still unclear (43). Our study demonstrates that JQF significantly increases the proportion of Th2 cells and negatively regulates interferon-γ production through the transcription factor Etv5. Koh et al. have shown that the ETS family transcription factor Etv5 regulates IL-10 production in Th2 cells (46), which may be crucial for the anti-inflammatory effects of Th2 cells. Additionally, Etv5 is considered a transcriptional suppressor of insulin secretion related to obesity and a novel transcription factor involved in regulating hepatic fatty acid metabolism (34, 35). Therefore, Etv5 may play an important role in JQF’s regulation of Th2 cells in hepatic steatosis and inflammation associated with NAFLD. Cell communication analysis further confirmed the key role of macrophage subgroups (KCs, MoMFs, NAM) and T cell subgroups (Th2 and Tc) in the treatment of NAFLD with JQF. After JQF treatment, overall cell communication was significantly enhanced, including an increase in the number and strength of inferred interactions. Additionally, the ICAM ligands acting on the macrophage subgroups and T cell subgroups were overall weakened after JQF treatment. This included a reduction in both the autocrine and paracrine signals of the macrophage subgroups, while the autocrine and paracrine signals of Th2 were enhanced, thereby reducing inflammation and alleviating fibrosis. Furthermore, after JQF treatment, TGFb signaling pathway cell communication was enhanced, especially in Th2, which also helps in anti-inflammatory and anti-fibrotic effects. Moreover, the global communication patterns also suggest that the communication patterns of certain cell groups changed following JQF treatment.

This study represents the first integration of scRNA-seq and ST to elucidate the immune characteristics of NAFLD and investigate the pharmacological mechanisms of JQF intervention. The novelty of our research lies in establishing experimental and computational foundations for exploring traditional Chinese medicine formulas and herbal medicines for the treatment of NAFLD, particularly from an immunomodulatory perspective. Additionally, our findings provide crucial insights and guidance for further exploration of the pharmacologically active components and clinical applications of JQF. However, this study has some limitations. Firstly, the JQF treatment was administered only at a clinically equivalent dose, without testing multiple dosages of traditional Chinese medicine, which will be addressed in future studies. Secondly, the sample size used for scRNA-seq and ST was relatively small, which may affect the stability of the results. In future studies, we will increase the sample size. Furthermore, we did not conduct additional experimental research to confirm the functional roles of key cell populations and targets. Despite these limitations, our research elucidates the potential of JQF to improve hepatic steatosis, inflammation, and fibrosis in NAFLD through immunomodulation, laying the foundation for more in-depth investigations into the mechanisms of JQF. In addition, we are diligently preparing for a randomized controlled trial of JQF. The goal is twofold: to secure robust evidence-based medical data regarding JQF’s efficacy in treating T2D, and to confirm the findings of this research using clinical samples, with the intention of offering novel therapeutic strategies and perspectives for T2D management.

## 5 Conclusion

In conclusion, our study confirms for the first time that JQF significantly ameliorates hepatic steatosis, inflammation, fibrosis, and glucose tolerance in NAFLD mice. Furthermore, by integrating scRNA-seq and ST, we demonstrated that the immune cell landscape in the liver undergoes a a significant reshaping during NAFLD and NASH, with notable alterations in monocytes, macrophages, and T cells. We observed that regions with pronounced immune cell infiltration exhibited the most severe liver inflammation and fibrosis, and that JQF significantly modulates these immune responses. Subsequent subcluster analysis and cell communication analysis identified specific macrophage subclusters (KCs and MoMFs) and T cell subclusters (Tc and Th2) as crucial cellular populations involved in the therapeutic effects of JQF. Additionally, SCENIC analysis elucidated the key upstream TFs regulated by JQF. Despite its therapeutic potential, the mechanisms by which JQF addresses NAFLD are complex and warrant additional comprehensive and rigorous research for a complete understanding. Our subsequent research will focus on the key cellular subpopulations and transcription factors identified as integral to JQF’s therapeutic efficacy. This focus is aimed at developing novel therapeutic approaches for the treatment of NAFLD.

## Data Availability

The datasets presented in this study can be found in online repositories. The names of the repository/repositories and accession number(s) can be found below: https://www.ncbi.nlm.nih.gov/, GSE270583 https://www.ncbi.nlm.nih.gov/, GSE270584.
